# Eating disorder cognitions: a comparison between Avoidant/Restrictive Food Intake Disorder (ARFID) and Anorexia Nervosa

**DOI:** 10.1186/s40337-025-01341-8

**Published:** 2025-11-13

**Authors:** H. Wilkinson, A. Carbonnier, H. Wilkinson-Herbots, M. Cooper

**Affiliations:** 1 Isis Education Centre, Oxford Institute of Clinical Psychology Training and Research, Warneford Hospital, Headington, Oxford, UK; 2 Department of Experimental Psychology, Medical Sciences Division, University of Oxford, Oxford, UK; 3 Oxford Health NHS Foundation Trust, Oxford, UK; 4 Berkshire Health NHS Foundation Trust, Berkshire, UK; 5 Royal College of Psychiatrists, London, UK; 6 Department of Statistical Science, University College London (UCL), London, UK

**Keywords:** Avoidant/Restrictive Food Intake Disorder, ARFID, Anorexia Nervosa, Eating disorders, Overvaluation of shape/weight, Diagnosis, Assessment, CBT, Cognitions, Beliefs

## Abstract

**Background:**

This study aims to investigate whether individuals with Avoidant/Restrictive Food Intake Disorder (ARFID) experience unhelpful cognitions that overlap with Anorexia Nervosa (AN). It also examines whether these cognitions play a role in driving problematic eating behaviours that are typically associated with AN because they are designed to prevent weight gain.

**Methods:**

There were 184 participants (68 individuals with AN, 61 individuals with ARFID, and 55 people with no eating disorder) who were screened using Diagnostic Statistical Manual (DSM-5) criteria. Participants were an adult community sample who completed an anonymous online survey. Questionnaires measured core beliefs, assumptions and automatic thoughts that are associated with AN, and an assessment of concerns about weight and shape was completed. An observational design was used to compare how responses varied according to diagnosis.

**Results:**

Individuals with ARFID were found to have significantly higher levels of disordered core beliefs, assumptions, automatic thoughts, and weight/shape concerns than people with no eating disorder. They showed lower levels of these cognitions relative to individuals with AN. Disordered assumptions and automatic thoughts explained a large proportion of variance in behaviours intended to prevent weight gain in this group.

**Conclusions:**

The findings have implications for the diagnosis and treatment of ARFID. They challenge diagnostic conceptualisations of ARFID as entirely separable from other eating disorders and any associated weight/shape concerns. They also highlight the need for clinicians to assess and treat unhelpful cognitions that may be maintaining disordered patterns of eating. Future research directions are discussed.

## Abstract (plain language summary)

Avoidant/Restrictive Food Intake Disorder (ARFID) is a new diagnosis that describes restricted eating caused by sensitivity to the qualities of food (e.g. texture), fear of bad consequences to eating (e.g. choking), or lack of appetite. The diagnostic criteria state that ARFID is entirely separable from other eating disorder diagnoses that are driven by body image concerns, such as Anorexia Nervosa (AN). There is limited research about whether people with ARFID experience unhelpful beliefs that play a role in their restricted eating.

In our study, we compare the beliefs of people with ARFID, with AN, and people with no eating disorders. We show that people with ARFID experience disordered cognitions and weight/shape concerns that are usually associated with AN. We also find that some of these cognitions are linked to behaviours designed to prevent weight gain in people with ARFID. This research is important because it challenges current conceptualisations of ARFID as fully distinct from other eating disorders, which has implications for assessment and diagnosis. It also raises the question of whether clinical interventions need to focus on targeting unhelpful beliefs that might be maintaining disordered eating behaviours. We discuss directions for future research.

## Background

Avoidant/Restrictive Food Intake Disorder (ARFID) is a relatively new diagnosis that was introduced in the 2013 edition of the Diagnostic Statistical Manual (DSM-5) [1]. It represents restrictive eating caused by sensitivity to sensory characteristics (e.g. texture), lack of interest, or fear of aversive consequences (e.g. choking). It is estimated to affect 0.3–15.5% of the general population [2]. ARFID can have major physical health consequences by causing nutritional deficiencies [3] and can severely impact people’s quality of life [4, 5].

Research has focused on how ARFID might differ from classical eating disorders (EDs), particularly anorexia nervosa (AN), which similarly involves restrictive eating. The DSM-5 states that AN and ARFID are distinct conditions that are distinguished by the presence of body image concerns [1]. In AN, weight or shape concerns represent the main driver of restriction. By contrast, much has been made of the absence of these concerns in ARFID and indeed this is one of the diagnostic criteria. Yet, this account has been challenged. Becker et al. [6] explained that their eating disorder team has observed several adolescent girls with ARFID (e.g. with choking phobia, or high selectivity for certain textures) who have simultaneously had AN or subsequently gone on to develop this disorder. Forbush et al. [7] stated that approximately 10% of patients in their ARFID clinic presented with clinically significant dissatisfaction with their weight or shape (see also 8–10).

**Fig. 1:**
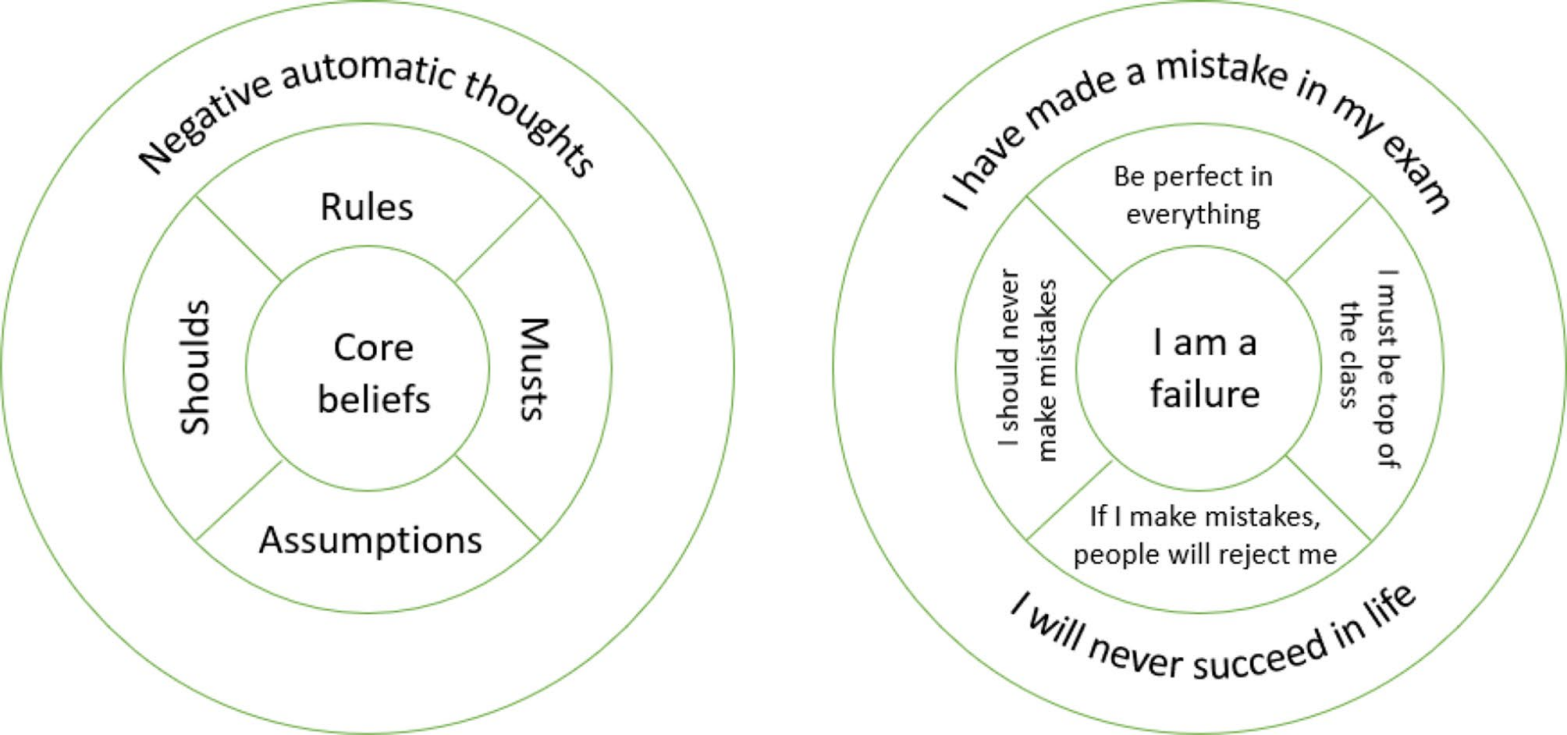
An illustration of Beck’s cognitive model of emotional disorders and an example of this for a patient. Adapted from Branch & Willson [80]

The presence of body image concerns has implications for diagnosis. It also raises the question of how far people with ARFID have unhelpful cognitions associated with other EDs, which may be a target for treatment. The first-line treatment for classical EDs is cognitive behavioural therapy (CBT). It works by addressing unhelpful behaviours and cognitions that are maintaining the disorder. In CBT, cognitions are conceptualised at three levels: core beliefs, assumptions and automatic thoughts [11]. Core beliefs are deep-seated beliefs people have about themselves and the world [12]. They give rise to assumptions, demands and ‘rules for living’, which in turn, shape people’s automatic thoughts in a specific situation. This is illustrated in Fig. 1. Negative core self-beliefs play a causal role in classical EDs [13, 14], and it has been argued that lasting recovery requires modifying them [15, 16]. Cognitive CBT techniques involve identifying distorted thinking patterns (e.g. catastrophising) and gathering evidence to challenge and reframe them.

Psychological treatments for ARFID are still in the phase of being piloted and tested and the evidence-base is very sparse [17, 18]. However, they also tend to favour a CBT approach, albeit with more of a behavioural focus employing strategies such as regular eating, graded exposure to novel foods, relaxation and behavioural experiments, rather than any thought-challenging techniques [19–21]. This perhaps reflects that there has been limited research on whether unhelpful cognitions play a role in ARFID and on their specific content.

In this study, we aim to address this gap by determining the degree to which people with ARFID overlap in experiencing core beliefs, assumptions and automatic thoughts that are commonly found in patients with classical EDs (referred to as ‘classical ED-related cognitions’). In addition, we explore whether these cognitions play a role in maintaining behaviours to prevent weight gain (referred to as ‘restraint’, see definition under Measures section). There are several grounds for our predictions. As discussed above, body image concerns appear to be present in some individuals with ARFID. Moreover, ARFID is associated with several factors which are prominent in classical EDs, including fear of negative evaluation [22], emotional dysregulation [23], high levels of adverse childhood events [24], and some parental behaviours [25]. In classical EDs, these events have been linked to unhelpful assumptions and core beliefs [16, 26–28], and thus this raises the question whether these cognitions are also present in ARFID. This study compares people with ARFID, AN and healthy controls (HC) in order to examine the hypotheses below:

### Primary hypotheses

1. It is predicted that the highest levels of classical ED-related core beliefs will be found in individuals with AN, followed by those with ARFID, followed by HC (i.e., people with AN will show significantly higher levels of classical ED-related core beliefs than both people with ARFID and HC, and people with ARFID will show significantly higher levels of these classical ED core beliefs than HC).
2. Individuals with AN will show the highest levels of classical ED-related assumptions, followed by individuals with ARFID, followed by HC.
3. Individuals with AN will show the highest levels of classical ED-related automatic thoughts, followed by individuals with ARFID, followed by HC.

If the data does not falsify these hypotheses, then further predictions can be made.

### Secondary hypotheses

1. Individuals with AN will show the highest levels of weight/shape concerns, followed by those with ARFID, followed by HC.
2. Restraint will be positively related to classical ED-related cognitions in people with ARFID.

## Method

### Design

An observational design was used to compare questionnaire responses across three independent groups: people with ARFID, AN and HC. Ethical approval was obtained from the Central University Research Ethics Committee at the University of Oxford (ref: R88894/RE001). Three experts-by-experience were involved in designing advertising material, information and consent forms, the survey, and debrief page.

### Participants

ARFID pathways in national healthcare services are few/non-existent and therefore participants were recruited from a community sample. Between March and August 2024, advertisements were shared through social media (Facebook and Instagram) and relevant mental health charities (Student Minds, Participate-MQ, ARFID Awareness UK). Participants had to be aged over 18 years and living in the UK, USA, Canada, Australia or New Zealand. They were excluded if they did not belong to one of the above three groups, and/or reported losing weight because of medical illness or a reason other than avoidance or restriction.

### Measures

The survey consisted of various self-report questionnaires assessing ED diagnosis, classical ED-related cognitions, and sample characteristics (see Table 1). Two of the Eating Disorder Beliefs Questionnaire (EDBQ) subscales (‘weight and shape as a means to acceptance by others’ and ‘weight and shape as a means to self-acceptance’) were used to assess the concept of ‘weight/shape concerns.’ Four items of the Eating Disorder Diagnostic Scale (EDDS-V) were summed to yield the total number of times that people engaged in various disordered eating behaviours over the past three months, as an index of ‘restraint.’ These behaviours were fasting, laxative/diuretic use, purging, and more intensive exercising specifically with the aim of counteracting the effects of eating or preventing weight gain.

**Table 1:** Outcome measures used within the study

Domain	Measure
**Grouping Variables**	
Classical eating disorder diagnosis	The DSM-5 version of the Eating Disorder Diagnostic Scale (EDDS-V) is a 23-item self-report measure that diagnoses anorexia nervosa, bulimia nervosa and binge eating disorder from healthy controls [29]. It represents an update of the original EDDS [30], but has not yet been validated. However, the original version demonstrates good test-retest reliability, internal consistency, criterion validity and convergent validity [31, 32].
ARFID diagnosis	The Pica, ARFID and Rumination Disorder Questionnaire (PARDI-AR-Q) [33] is a 32 item self-report questionnaire. It contains a 7-point Likert scale to measure common ARFID features including sensory-based avoidance, lack of interest, and fear of aversive consequences of food or eating. It has been validated and shows high specificity [34].
**Primary Outcome Variables**	
Classical ED-related core beliefs	The Eating Disorder Core Beliefs Questionnaire - Revised (ED-CBQ-R) [35, 36] is designed to capture common core beliefs in patients with classical eating disorders, such as anorexia nervosa and bulimia nervosa. This 15-item scale assesses four dimensions of eating disorder core beliefs of self-loathing, unassertive, demanding, and abandoned. Belief in each item is rated on a 7-point Likert scale from ‘feels very much untrue’ to ‘feels very much true.’ The subscales possess adequate internal consistency, and convergent and divergent validity [37].
Classical ED-related assumptions	The Eating Disorder Beliefs Questionnaire (EDBQ) [38] measures dysfunctional assumptions in classical eating disorder patients. It contains 22 items rated on a Likert (0-100) scale with endpoints anchored at “I do not usually believe this at all” and “I am usually completely convinced that this is true.” These map onto four subscales relating to negative self-beliefs, weight and shape as a means of acceptance by others, weight or shape as a means of self-acceptance, and control over eating. It possesses good validity and reliability [38, 39].
Classical ED-related automatic thoughts	The Thoughts Questionnaire (TQ) [40] is a 26-item scale that captures automatic thoughts about eating in classical eating disorder patients. Belief in each item is rated on a Likert (0-100) scale, with statements “I do not usually believe this at all” and “I am usually completely convinced that this is true” marking each end of the continuum. The TQ comprises three subscales relating to positive, negative and permissive thoughts about eating. The subscales demonstrate good internal consistency, construct validity and discriminant validity [40].
**Sample characteristics**	
Demographic details	Demographic questions relating to age, sex, gender identification, race, ethnicity and occupation were derived from the Office for National Statistics census, which was developed through research testing and user-engagement [41].
Depression	The Patient Health Questionnaire-8 (PHQ-8) [42] is an 8-item assessment of depression symptoms. It is derived from the PHQ-9 but omits an item that asks about suicide and suicidal ideation. Removing this item was required by ethical research governance since the study was anonymous and hence it would not be possible to provide support to individuals flagged as at risk of suicide. In this questionnaire, the frequency of experiencing various depressive symptoms over the past two weeks is rated on a four-point Likert scale, ranging from “not at all” to “nearly every day.” It demonstrates adequate reliability, sensitivity, specificity, internal consistency, and cross-country equivalence [43, 44].
Generalised Anxiety	The Generalised Anxiety Disorder Assessment (GAD-7) [45] is a 7-item screening tool and severity measure. Even though it was originally developed to assess generalised anxiety disorder, it is increasingly used as a general measure of anxiety [46] and anxiety disorder [47]. In this questionnaire, the frequency of experiencing various anxiety symptoms over the past two weeks is rated on a four-point Likert scale, ranging from “not at all” to “nearly every day.” It displays excellent convergent validity and internal consistency [48] and is reliable and valid in general population samples [49, 50].

### Procedure

Participants were invited to follow a link which presented them with an information sheet and consent form. Eligible participants were then able to complete the study which involved an anonymous online survey. Participants were required to complete a ‘Completely Automated Public Turing test to tell Computers and Humans Apart’ (CAPTCHA) as a security measure to deter bot attacks and spam and ensure the user was human. Participants who declined consent were not able to proceed. The final page of the survey contained a statement reminding participants of their self-worth and provided an optional set of items where participants could select their values, identify motivational quotes, and engage in guided imagery. There was also a list of support services. Participants were informed that the study was seeking to understand the similarities and differences between the beliefs that people with AN and ARFID held about themselves and about food. There was no financial reward for participation.

### Data analysis plan

#### Power

An a-priori power analysis was performed using Cohen’s Power Primer (1992). This calculated a minimum required sample size of *N* = 156 to detect a medium-sized difference (*d = 0.50*) across three independent groups with *0.80* power.

#### Data preparation

Prior to conducting any analyses, participants were classified into groups of AN, ARFID and HC using DSM-5 diagnostic criteria. The EDDS-V was used to identify participants who had a clinical diagnosis of AN and screen out participants with bulimia nervosa and binge eating disorder. The PARDI-AR-Q identified participants with ARFID. People with no clinical eating disorder were HC. In line with DSM-5 criteria, it was assumed that people cannot have a dual diagnosis of AN and ARFID, with AN functioning as the primary diagnosis.

#### Data checking

To prevent fraud, participants with duplicate Internet Protocol (IP) addresses were also excluded. Data validation was inbuilt for the kinds of responses allowed (for example, alphabetical text was not permitted for answers requiring a numerical response and responses were limited within the range of the measure). Participants who were missing responses on questionnaire items were excluded from the dataset. Unfortunately, a technical error meant that the survey terminated prematurely for a number of participants and thus it was not possible to meaningfully calculate the precise extent of missing responses. Out of 492 adults who consented to participate, 289 were screened out due to incomplete responding, ineligibility (neither ED diagnosis nor HC), or the survey ending prematurely. A further 19 participants were excluded due to a primary bulimia or binge diagnosis, or having a physical health condition that could influence eating habits (e.g. Crohn’s disease). The next step involved checking all data for adherence to statistical assumptions for analyses. The majority of data for each group was normally distributed. However, there was an issue of a floor effect in the healthy group where, expectedly, they showed low levels of classical ED-related cognitions and weight/shape concerns. The decision was made to continue with parametric testing since Multivariate Analysis of Variance (MANOVA) is robust to violations of normality particularly when reporting Pillai’s Trace statistic [51], and the remaining data were suitable.

#### Identification of covariates

Demographic variables that differed significantly between AN, ARFID and HC participants were intended to be included as covariates in analyses of group differences (hypotheses 1–4). A one-way Analysis of Variance (ANOVA) showed that age was a significant covariate. However, categorical demographic variables (sex, gender identification, race, ethnicity, occupation) were unsuitable for running Chi-Square tests since there were not enough data in each cell (*N* < 5).

#### Statistical tests

For the primary hypotheses, a one-way multivariate analysis of covariance (MANCOVA) aimed to analyse whether diagnostic groups showed different levels of classical ED-related cognitions (core beliefs, assumptions and thoughts), whilst controlling for age. Following this, post-hoc one-way analysis of covariance (ANCOVA) and Bonferroni corrected t-tests would explore any significant effects. Plans for secondary analyses included a one-way MANCOVA to compare group differences in weight/shape concerns, with age as a covariate. Linear regression analyses aimed to test whether classical ED-related cognitions predicted ‘restraint’ behaviours designed to prevent weight gain in people with ARFID.

## Results

### Sample characteristics

The final dataset included 184 participants assigned to groups of AN (*N = 68*), ARFID (*N = 61*) and HC (*N = 55*). Table 2 shows sample characteristics organised by group. Overall, most participants were White (87%) and reported female sex (94%) and gender identification (79%). Approximately two thirds were in some form of paid work (66%). The overall mean age of participants was 28.50 years, however age varied significantly across the three groups, *F*_(2, 154)_ = 9.80, *p* = < 0.001. HC participants were significantly older than AN (*p* <.001) and ARFID participants (*p* =.001), whereas there was no significant difference between AN and ARFID participants (*p* = 1.000). Body mass index (BMI) also varied significantly across the three groups (*F*_(2, 181)_ = 36.38, *p* = < 0.001) where, consistent with diagnosis, AN participants had a significantly lower BMI than the other two groups (*p* <.001 for both). BMI was in the underweight range for AN participants and in the normal range for ARFID and HC participants.

Group-level differences existed in self-reported symptoms of anxiety, *F*_(2, 181)_ = 47.89, *p* = < 0.001, and depression, *F*_(2, 181)_ = 52.01, *p* <.001. The AN group reported the highest levels of anxiety compared to the ARFID group (*p* =.042), who in turn, had higher levels of anxiety than HC (*p <*.001). The same trend was seen for depressive symptoms whereby AN participants had significantly more symptoms than ARFID participants (*p =*.006), who in turn, had more symptoms than HC (*p <*.001).

**Table 2:** Sample characteristics organised by group

Characteristics	AN	ARFID	Healthy	Total
Ethnicity (N, %)				
Asian/Asian British	6 (8.8)	2 (3.3)	2 (3.6)	10 (5.4)
Black/African/Caribbean/Black British	1 (1.5)	-	3 (5.5)	4 (2.2)
Mixed/Multiple ethnic groups	2 (2.9)	3 (4.9)	2 (3.6)	7 (3.8)
Other ethnic group	-	2 (3.3)	1 (1.8)	3 (1.6)
White	59 (86.8)	54 (88.5)	47 (85.5)	160 (87.0)
Sex (N, %)				
Female	66 (97.1)	59 (96.7)	48 (87.3)	173 (94.0)
Male	2 (2.9)	2 (3.3)	7 (12.7)	11 (6.0)
Gender identification (N, %)				
Female	58 (85.3)	41 (67.2)	46 (83.6)	145 (78.8)
Genderfluid	**-**	2 (3.3)	-	2 (1.1)
Male	**-**	2 (3.3)	7 (12.7)	9 (4.9)
Non-binary	8 (11.8)	13 (21.3)	2 (3.6)	23 (12.5)
Not specified	1 (1.5)	1 (1.6)	-	2 (1.1)
Transgender	1 (1.5)	2 (3.3)	-	3 (1.6)
Occupation (N, %)				
In paid work (employee or self-employed)	36 (52.9)	26 (42.6)	46 (83.6)	108 (58.7)
In paid work taking temporary leave	8 (11.8)	4 (6.6)	1 (1.8)	13 (7.1)
Long term sick or disabled	7 (10.3)	5 (8.2)	2 (3.6)	14 (7.6)
Looking after family or home	-	2 (3.3)	2 (3.6)	4 (2.2)
Other	3 (4.4)	2 (3.3)	-	5 (2.7)
Retired	-	2 (3.3)	-	2 (1.1)
Student	14 (20.6)	20 (32.8)	4 (7.3)	38 (7.1)
Age (M, SD)				
	25.66 (7.80)	26.58 (9.93)	34.26 (2.02)	28.50 (11.13)
BMI (M, SD)				
	16.25 (1.81)	23.47 (7.80)	22.86 (4.89)	20.62 (6.29)
Total anxiety scores (M, SD)				
	15.21 (5.13)	12.85 (5.58)	5.91 (5.42)	11.64 (6.60)
Total depressive scores (M, SD)				
	16.37 (5.65)	13.15 (6.16)	5.69 (5.75)	12.11 (7.31)

Sample characteristics are shown for the AN, ARFID and healthy control groups and at an overall level. Number (N) and percentage (%) of participants are reported for categorical data. Means (M) and standard deviations (SD) are reported for continuous data

Of note, on the GAD-7 measure of anxiety symptoms, a total score of 0–4 indicates minimal anxiety, 5–9 indicates mild anxiety,10–14 indicates moderate anxiety, and greater than 15 indicates severe anxiety [45]

On the PHQ-8 measure of depressive symptoms, a score of 0–4 is rated as normal, 5–9 as mild, 10–14 as moderate, and 15–19 as moderately severe, and 20–24 as severe [42]

### Internal consistency of scales

Cronbach’s reliability showed good to excellent consistency for the complete ED-CBQ-R, EDBQ, TQ questionnaires and the two EDBQ subscales assessing weight/shape concerns (*α* = 0.82–0.98). This is important as previous research has only validated individual subscales of the ED-CBQ-R, EDBQ and TQ, whereas the tests of the primary hypotheses were not adequately powered to detect differences at this level.

### Primary hypotheses: presence of classical ED-related cognitions

**Fig. 2:**
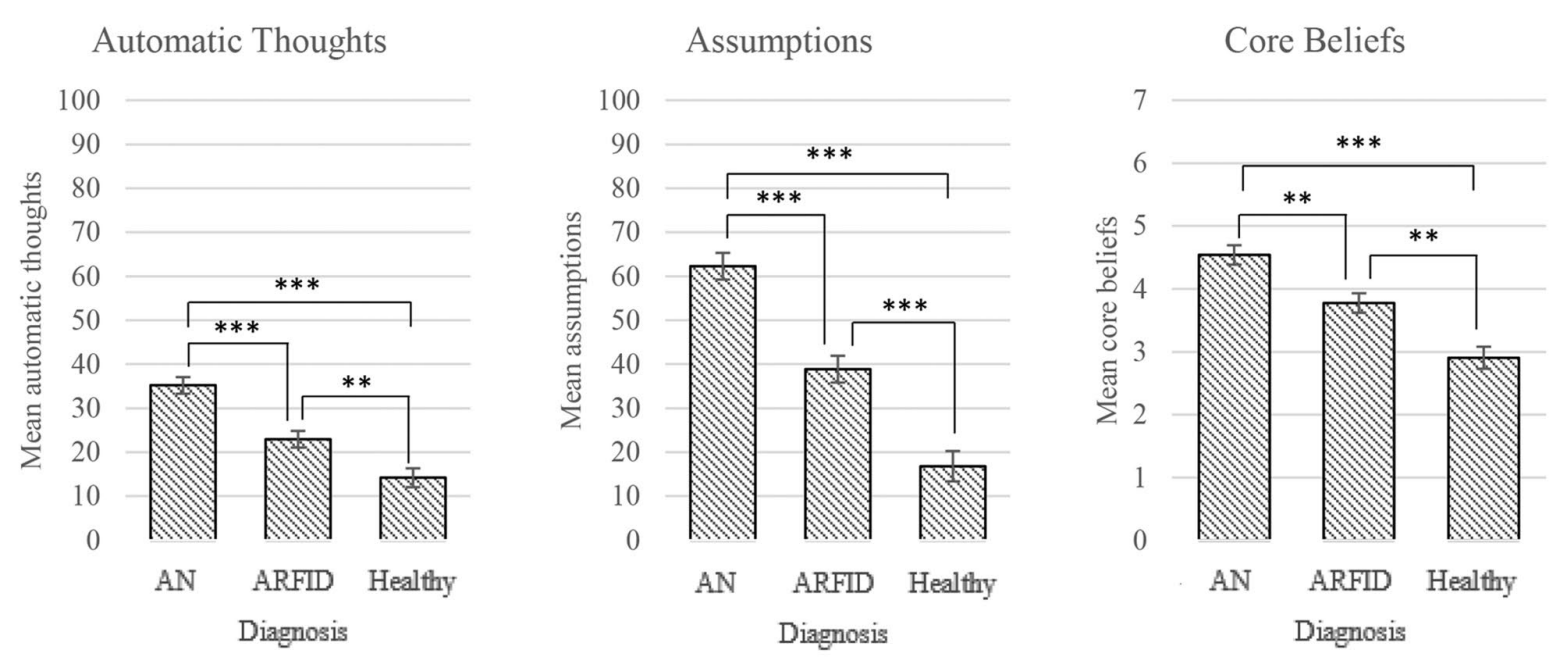
Graphs depict estimated marginal means and standard errors for scores on the TQ (automatic thoughts), EDBQ (assumptions) and ED-CBQ-R (core beliefs). This is shown for each group (AN, ARFID and healthy controls). Significant mean differences are marked such that one asterisk (*) denotes a value of *p* <.050 (**), two asterisks denote *p* <.010, and three asterisks (***) denote *p* <.001

A one-way MANCOVA was conducted to investigate whether people with AN, ARFID and HC showed different levels of classical ED-related core beliefs, assumptions and thoughts, whilst controlling for age. The analysis revealed a significant overall effect of diagnostic group on classical ED-related cognitions, *F*_(6, 304)_ = 12.61, *p* <.001, *η*^*2*^ = 0.20. Univariate tests confirmed that there were group differences in classical ED-related core beliefs, *F*_(2, 153)_ = 23.51, *p* <.001, *η*^*2*^ = 0.26; assumptions, *F*_(2, 153)_ = 47.64, *p* <.001, *η*^*2*^ = 0.38; and thoughts, *F*_(2, 153)_ = 26.99, *p* <.001, *η*^*2*^ = 0.26. Pairwise comparisons revealed a pattern whereby people with AN had significantly more classical ED-related core beliefs, assumptions and thoughts than people with ARFID, who in turn, had significantly more of these cognitions than HC. Estimated marginal means are shown graphically in Fig. 2 (see Table A1 in the appendix for the results).

### Secondary hypotheses

#### Prevalence of weight/shape concerns

**Fig. 3:**
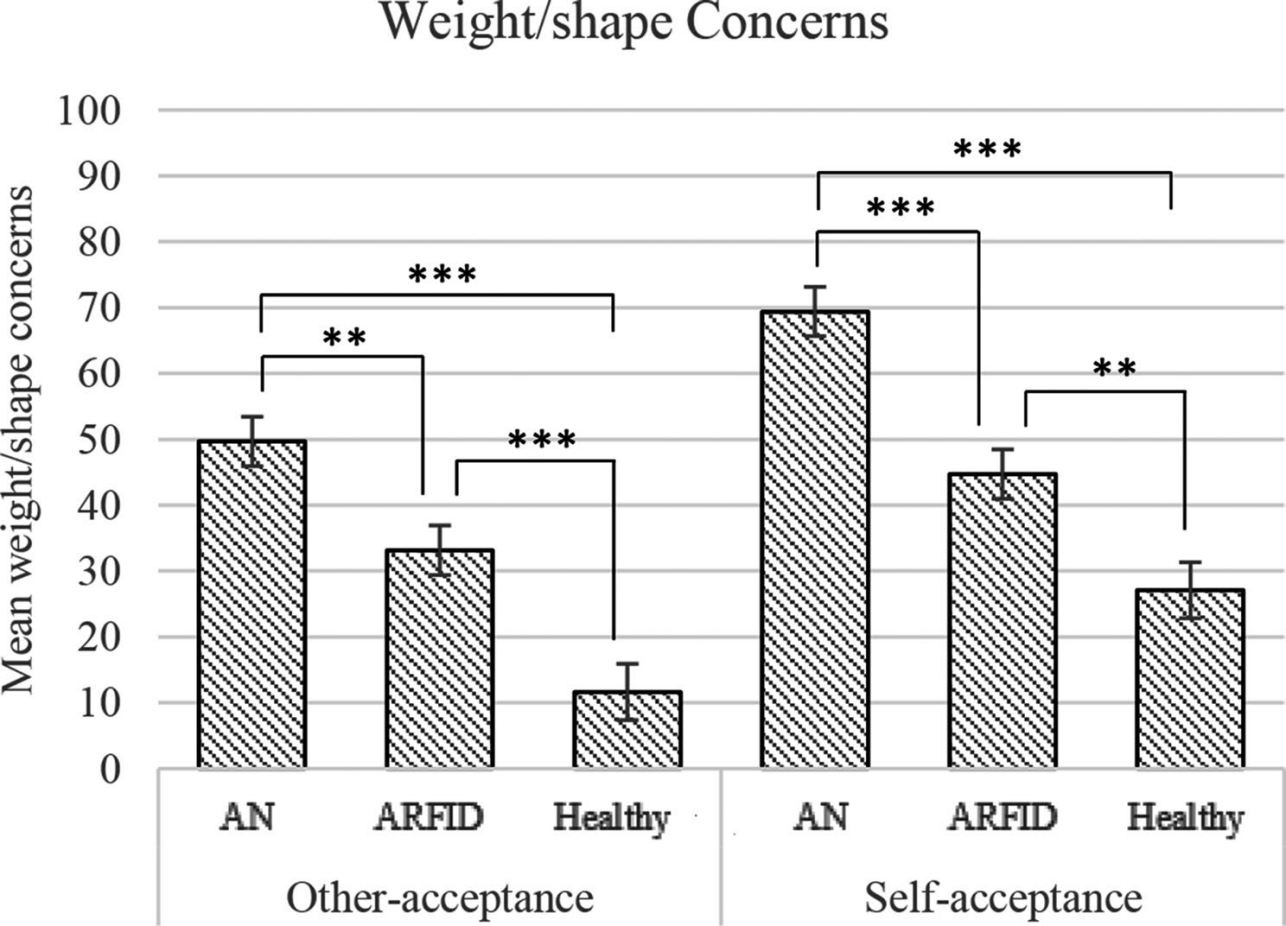
Graphs depict estimated marginal means and standard errors for scores on the two EDBQ subscales of weight/shape concerns as a means of acceptance by others (left graph) and weight/shape concerns as a means of self-acceptance (right graph). This is shown for each group (AN, ARFID and healthy controls). Significant mean differences are marked such that one asterisk (*) denotes a value of *p* <.050 (**), two asterisks denote *p* <.010, and three asterisks (***) denote *p* <.001

To investigate whether there were differences in weight/shape concerns among the diagnostic groups, a one-way MANCOVA was performed. The analysis revealed a significant overall effect of diagnostic group on weight/shape concerns, *F*_(4, 306)_ = 13.40, *p* <.001, *η*^*2*^ = 0.15. Univariate tests confirmed that there were group differences in both EDBQ subscales relating to weight/shape as a means of acceptance from others, *F*_(2, 153)_ = 21.41, *p* <.001, *η*^*2*^ = 0.22; and weight/shape as a means of self-acceptance, *F*_(2, 153)_ = 27.90, *p* <.001, *η*^*2*^ = 0.27. Pairwise comparisons revealed the same trend as previously shown, whereby people with AN had significantly more ED-related weight/shape concerns, followed by people with ARFID, and then HC. Estimated marginal means shown in Fig. 3 (see Table A2 in the appendix for the results).

#### Cognitive predictors of restraint in ARFID

Table 3 shows a correlation matrix between the predictor variables and restraint. Classical ED-related assumptions and automatic thoughts had significant positive correlations with restriction. They were also significantly correlated with each other. Initially, a stepwise multiple regression had been planned to identify the most influential cognitive predictors of restriction in the ARFID group. However, this test was found to be unsuitable because suppression effects were observed, including a reversal paradox, whereby the association between one of the predictors and the outcome variable became negative when other predictors were added to the analysis. This artefact was likely due to the multicollinearity between the three predictor variables resulting in unstable regression estimates. Hence, three separate regression models were run to address this.

**Table 3:** Pearson’s correlation between variables for the ARFID group

Variable	Core beliefs	Assumptions	Thoughts	Restraint
Core beliefs	-			
Assumptions	0.56 (< 0.001)	-		
Thoughts	0.31 (0.014)	0.79 (< 0.001)	-	
Restraint	0.15 (0.235)	0.67 (< 0.001)	0.51 (< 0.001)	-

The linear regression model with core beliefs as a predictor was not found to be significant, *F*_(1, 59)_ = 1.44, *p* =.235. However, the regression model with assumptions as a predictor was statistically significant, *F*_(1, 59)_ = 46.91, *p* <.001. This model explained approximately 44.3% of the variance in restraint (adjusted *R*^*2*^). The fitted regression model was: number of restraint behaviours = -1.81 + 0.30 * EDBQ score. That is, for each one-point increase in the total EDBQ questionnaire score, there was a predicted 0.30 increase in restraint behaviours among people with ARFID. The slope predicting restraint from EDBQ scores lay between 0.21 and 0.38 (95% confidence interval).

The regression model with automatic thoughts as a predictor was also significant, *F*_(1, 59)_ = 20.84, *p* <.001. This model explained approximately 24.9% of the variance in restraint (adjusted *R*^*2*^). The fitted regression model was: number of restraint behaviours = − 0.37 + 0.46 * TQ score; indicating that for each one-point increase in the TQ, restraint behaviours were predicted to increase by 0.46 among people with ARFID. The 95% confidence interval of the slope was between 0.26 and 0.66.

## Discussion

This study investigated whether people with ARFID experience unhelpful cognitions associated with classical eating disorders, and whether these cognitions predict behaviours aimed at preventing weight gain. In line with our hypotheses, people with ARFID showed significantly higher levels of classical ED-related core beliefs, assumptions and automatic thoughts than people with no eating disorder. They also showed significantly more weight/shape concerns than people with no eating disorder. Individuals with AN showed the very highest levels of classical ED-related cognitions and weight/shape concerns. A further finding was that, in the ARFID group, these classical ED-related assumptions and automatic thoughts explained a large proportion of the variance in restraint behaviours. To summarise, these results demonstrate that individuals with ARFID and classical ED patients have some shared experiences of unhelpful cognitions and weight/shape concerns. They also point to the significant role that cognitions may play in maintaining patterns of disordered eating in people with ARFID.

Of note, classical ED-related core beliefs did not significantly predict restraint. It has been argued that eating disorders occur through the “fusion” of negative core beliefs and assumptions, whereby people restrict food and engage in behaviours to compensate for these highly damaging beliefs (e.g. striving for thinness to feel less unworthy) [52, 53]. Thus, core beliefs alone may not be sufficient to predict restraint in ARFID. Alternatively, it may be that different core beliefs are related to restraint behaviours in ARFID compared to classical EDs.

In line with the DSM-5, we were required to place people who had an ARFID presentation in the AN group if they met the criteria for both disorders (AN is treated as the primary diagnosis). However, this process meant that individuals with high levels of disordered cognitions were likely omitted from the ARFID group. In light of this, our findings provide particularly strong support for the hypothesis that people with ARFID show higher classical ED-related cognitions and weight/shape concerns than healthy controls. It is also perhaps remarkable that the ARFID group showed this despite not being underweight (like the AN group).

Taken together, our findings add to a small but growing literature suggesting ARFID is not so distinct from classical EDs as the DSM-5 would suggest. There are several reports that ARFID and weight/shape concerns can co-occur [6–8, 54–58]. Indeed, a study by Jhe et al. [59] recently identified weight/shape concerns in over 250 youth with ARFID. A number of clinicians and researchers have proposed that there are three ARFID profiles depending on whether food restriction is driven by either sensory sensitivity, low appetite, or fear of aversive consequences (e.g. see 51). Whilst Jhe et al.’s study did not find that weight/shape concerns varied between different ARFID profiles or age, higher concerns were found in youth who were gender diverse and youth with overweight/obesity status [59]. Another recent study with adult participants also found a degree of overlap between ARFID and classical ED patients in their motivations to restrict food [60]. A subgroup of ED patients showed both ARFID-based (i.e., driven by sensory sensitivity, low appetite, or fear) and weight/shape-based motivations to eat less food. Indeed, several patients with ARFID diagnoses showed weight/shape motivations, and several patients with classical ED diagnoses showed ARFID-based motivations.

### Theoretical implications

Thus, taken together with other research, our results raise interesting questions about whether diagnostic refinement is needed [61]. Currently, the DSM-5 treats ARFID as an exception by presenting it as the only ED for which it is not possible to have a dual diagnosis with other EDs such as AN and bulimia nervosa [1]. However, it is unclear from a theoretical standpoint, why weight/shape concerns should be mutually exclusive to any sensory sensitivities, low appetite, or fear of aversive consequences around food. There appear to be increasing counterexamples to this in the literature. Our study was theoretically-informed in applying Beck’s cognitive model of emotional disorders [13]. This framework of core beliefs, assumptions and automatic thoughts underpins much of CBT. Indeed, CBT models of a wide range of clinical presentations are derived from this framework, including models of AN and bulimia nervosa [15, 16, 26, 62–64]. Current models of ARFID appear to be relatively limited in specifying the connections between disordered cognitions and behaviours. For example, Thomas and Eddy’s CBT manual depicts three models relating to each ARFID profile (see reference 65, p. 21–23). These models propose that “negative feelings and predictions about consequences of eating” mediate relationships from biological predispositions and/or food-related trauma, to food restriction, but they do not describe the specific content of these predictions, nor address how weight/shape concerns may also come into play. It is possible that individuals with ARFID experience cognitions specific to this disorder, or cognitions that are transdiagnostic with classical EDs, or that both of these co-exist. It may also be useful for future models to separate the different levels of cognition according to Beck’s cognitive framework rather than overlook this and treat all cognitions as the same [11].

### Clinical implications

Based on our findings, we recommend that clinicians routinely assess ARFID patients’ cognitions in line with Beck’s framework and screen for any weight/shape concerns [11]. Clinicians may need to target disordered cognitions within a patient’s formulation and treatment plan. The evidence that cognitions overlap between ARFID and classical EDs suggests that a transdiagnostic approach may be suitable for some ARFID patients, and that it may be useful to consider drawing on cognitive CBT techniques found in other ED treatments.

Of note, we observed that the ARFID group showed clinical levels of anxiety and depression. This is consistent with other research showing increased rates of comorbidity in ARFID [3, 66, 67] which are broadly comparable to other EDs [5, 68, 69]. Bryant-Waugh et al. have put forward an evidence-informed ARFID care pathway for children and adolescents, which recommends screening for other mental health disorders and neurodiversity as part of clinical assessments [17]. Our findings are consistent with this.

### Limitations and future research directions

Our study was not sufficiently powered to analyse subscale differences on the questionnaires measuring classical ED-related cognitions. It is possible that some subscales may be particularly characteristic of certain diagnoses, for example, AN patients have been found to score more highly on ‘negative self-beliefs’ than dieters and healthy controls [70]. It would be useful for future research to analyse which subscales relate most strongly to ARFID, which may support diagnosis and enable cognitive interventions to be more targeted.

It would also be useful to determine how patients’ pattern of responses may vary according to their ARFID profile, severity, and other clinical features such as comorbid depression, anxiety and autism. Indeed, great heterogeneity has been documented in clinical ARFID presentations [71–73] and it has been suggested that this may reflect different underlying mechanisms responsible for the onset and maintenance of ARFID [17]. Determining whether gender significantly influences participants’ responses would also be useful. In our study, the majority of participants were female which is a problem considering the well-established differences between females and males in measures of weight and shape concerns [74]. This study was limited to recruiting a community sample and diagnosing people based on self-report questionnaires (rather than clinical interview), because there are few specialist ARFID services for adults in the UK [75]. Published accounts of treatment-seeking families show that seeking help does not always result in appropriate referral [76–78]. Nevertheless, it would be beneficial for future research to replicate this study in patients with a formal diagnosis and determine whether the findings broadly generalise or only apply to certain patient subgroups.

Finally, it would be useful for future research to characterise and determine what drives the cognitions and behaviours that are specific to ARFID. The measures we used were developed with classical ED patients, and thus it is possible that ARFID patients demonstrate other core beliefs, assumptions and automatic thoughts that have not been captured here. Surveys, momentary sampling analysis, and phenomenological and thematic qualitative studies may reveal the content of ARFID patients’ cognitions. Interviews in which a qualified clinician uses the CBT questioning technique of ‘downward arrowing’ (see [79]) could enable further exploration of patients’ deeper meanings around food and eating, and determine assumptions and core beliefs linked to their eating difficulties. It would also be useful to explore how cognitions relate to behaviours characteristic of ARFID eating difficulties (e.g. restriction based on texture).

## Conclusions

This study presents novel findings that individuals with ARFID demonstrate core beliefs, assumptions, automatic thoughts and weight/shape concerns that are characteristic of classical eating disorders such as AN, and further, that some of these cognitions predict disordered eating behaviours aimed at preventing weight gain. These findings add to growing literature that challenges the DSM-5 conceptualisation of ARFID as distinct from classical eating disorders. These results also highlight a need for clinicians to assess whether unhelpful cognitions may be contributing to a patient’s eating difficulties and attend to this by using cognitive treatment techniques.

## Appendix

**Appendix 1**

Results of MANCOVA comparing levels of ED-related cognitions across individuals with AN, ARFID and HC.

**Table A1:** MANCOVA results comparing levels of ED-related cognitions across groups

Dependent variable	(I) Diagnosis	Mean	Std. error	(J) Diagnosis	Mean diff. (I-J)	Std. error	Sig.
Thoughts	AN	35.19	1.88	ARFID	12.24	2.64	< 0.001
				Healthy	20.99	2.92	< 0.001
	ARFID	22.95	1.89	AN	-12.24	2.64	< 0.001
				Healthy	8.78	2.90	0.009
	Healthy	14.20	2.14	AN	-20.99	2.92	< 0.001
				ARFID	-8.78	2.90	0.009
Assumptions	AN	62.28	3.03	ARFID	23.39	4.25	< 0.001
				Healthy	45.45	4.69	< 0.001
	ARFID	38.89	3.04	AN	-23.39	4.25	< 0.001
				Healthy	22.06	4.66	< 0.001
	Healthy	16.83	3.44	AN	-45.45	4.69	< 0.001
				ARFID	-22.06	4.66	< 0.001
Core beliefs	AN	4.54	0.15	ARFID	0.76	0.22	0.002
				Healthy	1.64	0.24	< 0.001
	ARFID	3.78	0.16	AN	− 0.77	0.22	0.002
				Healthy	0.87	0.24	0.001
	Healthy	2.91	0.18	AN	-1.67	0.24	< 0.001
				ARFID	− 0.87	0.24	0.001

*‘Mean’ refers to the estimated marginal means with the covariate of age being evaluated in the model at 28.50 years*,* and ‘std. error’ refers to their standard error. ‘Mean diff.’ represents the mean difference between two groups of estimated marginal means. ‘Sig.’ refers to the Bonferroni-adjusted p-value.*

**Appendix 2**

Results of MANCOVA comparing weight/shape concerns across individuals with AN, ARFID and HC

**Table A2:** MANCOVA results comparing levels of weight/shape concerns across groups

Dependent variable	(I) Diagnosis	Mean	Std. error	(J) Diagnosis	Mean diff. (I-J)	Std. error	Sig.
Weight/shape as a means of acceptance from others	AN	49.68	3.76	ARFID	16.51	5.27	0.006
				Healthy	38.03	5.81	< 0.001
	ARFID	33.17	3.76	AN	-16.51	5.27	0.006
				Healthy	21.52	5.78	< 0.001
	Healthy	11.65	4.27	AN	-38.03	5.81	< 0.001
				ARFID	-21.52	5.78	< 0.001
Weight/shape as a means of self-acceptance	AN	69.40	3.74	ARFID	24.67	5.24	< 0.001
				Healthy	42.32	5.78	< 0.001
	ARFID	44.73	3.74	AN	-24.67	5.24	< 0.001
				Healthy	17.64	5.75	0.008
	Healthy	27.09	4.25	AN	-42.32	5.78	< 0.001
				ARFID	-17.64	5.75	0.008

*‘Mean’ refers to the estimated marginal means with the covariate of age being evaluated in the model at 28.50 years*,* and ‘std. error’ refers to their standard error. ‘Mean diff.’ represents the mean difference between two groups of estimated marginal means. ‘Sig.’ refers to the Bonferroni-adjusted p-value.*

## Data Availability

The data that support the findings of this study are available from the corresponding author, HWH , upon reasonable request.

## Abbreviations

AN: Anorexia Nervosa
ANOVA: Analysis of Variance test
ANCOVA: Analysis of Covariance test
ARFID: Avoidant/Restrictive Food Intake Disorder
BMI: Body Mass Index
BN: Bulimia Nervosa
CAPTCHA: Completely Automated Public Turing test to tell Computers and Humans Apart
CBT: Cognitive Behavioural Therapy
Classical eating disorders: eating disorders that are driven by weight/shape concerns and which featured in previous editions of the DSM-5
Classical ED-related cognitions: core beliefs, assumptions and automatic thoughts that are commonly found in patients with classical eating disorders
DSM-5: Diagnostic Statistical Manual Fifth Edition (DSM-5)
ED: Eating disorder
EDBQ: The Eating Disorder Beliefs Questionnaire
ED-CBQ-R: The Eating Disorder Core Beliefs Questionnaire - Revised
EDDS-V: The Eating Disorder Diagnostic Scale
GAD-7: The Generalised Anxiety Disorder Assessment
HC: Healthy control i.e., an individual without any eating disorder
MANOVA: Multivariate Analysis of Variance test
PARDI-AR-Q: The Pica, ARFID and Rumination Disorder Questionnaire
PHQ-8: The Patient Health Questionnaire– 8 item version
Restraint: Behaviours to prevent weight gain
TQ: The Thoughts Questionnaire
